# Supplementary material for: Existing gaps and missed opportunities in delivering quality nutrition services in primary healthcare: a descriptive analysis of patient experience and provider competence in 11 low-income and middle-income countries
Source: BMJ Open. 2023 Feb 28;13(2):e064819. doi: 10.1136/bmjopen-2022-064819 (PMC9980366; doi:10.1136/bmjopen-2022-064819)
Supplement: Author's manuscript [file bmjopen-2022-064819.draft_revisions.pdf]

BMJ Open is committed to open peer review. As part of this commitment we make the peer review history of every article we publish publicly available.

When an article is published we post the peer reviewers' comments and the authors' responses online. We also post the versions of the paper that were used during peer review. These are the versions that the peer review comments apply to.

The versions of the paper that follow are the versions that were submitted during the peer review process. They are not the versions of record or the final published versions. They should not be cited or distributed as the published version of this manuscript.

BMJ Open is an open access journal and the full, final, typeset and author-corrected version of record of the manuscript is available on our site with no access controls, subscription charges or pay-per-view fees (http://bmjopen.bmj.com).

If you have any questions on BMJ Open's open peer review process please email
info.bmjopen@bmj.com

BMJ Open

Quality of Nutrition Services in Primary Healthcare: Existing Gaps and Missed Opportunities in Patient Experience and Provider Competence in 11 Low and Middle-Income Countries

Journal:	*BMJ Open*
Manuscript ID	bmjopen-2022-064819
Article Type:	Original research
Date Submitted by the Author:	18-May-2022
Complete List of Authors:	Ramadan, Marwa; World Bank Group; Alexandria University Faculty of Medicine<br>Muthee, Tonny B; World Bank Group<br>Okara, Latifat; World Bank Group<br>Feil, Cameron; World Bank Group<br>Villar-Uribe , Manuela; World Bank Group
Keywords:	Nutrition < TROPICAL MEDICINE, PUBLIC HEALTH, PRIMARY CARE, Quality in health care < HEALTH SERVICES ADMINISTRATION & MANAGEMENT

SCHOLARONE™
Manuscripts

I, the Submitting Author has the right to grant and does grant on behalf of all authors of the Work (as defined in the below author licence), an exclusive licence and/or a non-exclusive licence for contributions from authors who are: i) UK Crown employees; ii) where BMJ has agreed a CC-BY licence shall apply, and/or iii) in accordance with the terms applicable for US Federal Government officers or employees acting as part of their official duties; on a worldwide, perpetual, irrevocable, royalty-free basis to BMJ Publishing Group Ltd ("BMJ") its licensees and where the relevant Journal is co-owned by BMJ to the co-owners of the Journal, to publish the Work in this journal and any other BMJ products and to exploit all rights, as set out in our licence.

The Submitting Author accepts and understands that any supply made under these terms is made by BMJ to the Submitting Author unless you are acting as an employee on behalf of your employer or a postgraduate student of an affiliated institution which is paying any applicable article publishing charge ("APC") for Open Access articles. Where the Submitting Author wishes to make the Work available on an Open Access basis (and intends to pay the relevant APC), the terms of reuse of such Open Access shall be governed by a Creative Commons licence – details of these licences and which Creative Commons licence will apply to this Work are set out in our licence referred to above.

Other than as permitted in any relevant BMJ Author's Self Archiving Policies, I confirm this Work has not been accepted for publication elsewhere, is not being considered for publication elsewhere and does not duplicate material already published. I confirm all authors consent to publication of this Work and authorise the granting of this licence.

For peer review only

Quality of Nutrition Services in Primary Healthcare: Existing Gaps and Missed Opportunities in Patient Experience and Provider Competence in 11 Low and Middle-Income Countries

Authors and affiliations

Marwa Ramadan[1]*, Tonny B Muthee[1] , Latifat Okara[1], Cameron Feil[1], Manuela Villar Uribe[1]

[1]Health Nutrition and Population Global Practice, World Bank Group, Washington, DC, USA.

***Corresponding Author**

Marwa Ramadan

Health Nutrition and Population Global Practice, World Bank Group, Washington, DC, USA

mramadan@worldbank.org

Keywords

Primary health care; nutrition services; healthcare quality; measurement; Low- and Middle-income countries

Abstract

Objectives: To assess the competence of PHC providers in delivering maternal and child nutrition services at the PHC level and patients' experience in receiving the recommended components of care

Design: Observational cross-sectional analysis

Setting: Healthcare facilities in Low- and Middle-Income Countries (LMICs) with available service provision assessment surveys (Afghanistan (2018), Democratic Republic of Congo (2018), Haiti (2017), Kenya (2010), Malawi (2013-2014), Namibia (2009), Nepal (2015), Rwanda (2007), Senegal (2018), Tanzania (2015), and Uganda (2007).

Participants: 18644 antenatal visits and 23262 sick child visits in 8458 facilities across 130 subnational areas in 11 LMICs from 2007 to 2019.

Outcomes: 1) Provider competence assessed as the direct observations of actions performed during antenatal care and sick child visits; and 2) Patients 'experience defined as the self-reported awareness of the nutrition services received during ANC and Sick child visits and provider effectiveness in delivering these services

Results: Except for DRC, all countries scored below 50 % on patients' experience and provider competence. More than 80 % of clients were advised on taking iron supplements during pregnancy; however, less than 40 % of patients were advised on iron side effects in all the studied countries. Across all countries, providers commonly took anthropometric measurements of expectant mothers and children; however, such assessments were rarely followed up with advice or counseling about growth patterns. In addition, less than 20 % of observed providers advised on early/ immediate breastfeeding in all countries with available data.

Conclusion: The 11 assessed countries demonstrated the delivery of limited nutrition services; nonetheless, the apparent deficiency in the extent and depth of questions asked for the majority of tracer activities revealed significant opportunities for improving the quality of nutrition service delivery at the PHC level.

For peer review only

For peer review only - http://bmjopen.bmj.com/site/about/guidelines.xhtml

Strengths and limitations

- Existing literature focused on the availability of nutrition services through system inputs. However, this study bridges a gap by focusing on the service delivery processes that underpin the quality of nutrition services at the PHC level
- The methodology utilized in the present study demonstrates how standardized health facility surveys can be a valuable tool in capturing both the patient and the provider perspectives in delivering quality nutrition services
- The service provision assessment survey utilized in the present analysis only focused on facility-based nutrition services with inability to capture community-based nutrition services. The current versions of the survey also do not capture vital evidence-based nutrition interventions such as calcium, balanced energy, and protein, multiple micronutrient supplementation in pregnancy
- This study only assessed the quality of nutrition services in antenatal care and sick child visits, data were inadequate for other key PHC services as non-communicable diseases.

For peer review only

For peer review only - http://bmjopen.bmj.com/site/about/guidelines.xhtml

Background

Over the past 20 years, significant progress has been made in nutrition-related outcomes in many Low- and Middle-Income countries (LMICS). Many LMICs, however, now face the dual malnutrition burden of undernutrition and overweight, posing significant challenges for progress in nutrition, population health, and human capital development. Globally, it is estimated that one out of every nine people is hungry or undernourished and that one in every three people is overweight or obese (1,2). Furthermore, the food insecurity caused by the COVID-19 pandemic and ongoing conflicts such as in Ukraine and Russia are additional threats to nutrition-related progress. The United Nations estimates that 2.37 billion people lacked access to adequate food sources in 2020, an increase of 320 million people compared to 2019. The combined effects of the pandemic, and conflict-related turmoil if left unmitigated, will likely result in longstanding and severe consequences for health and nutrition (3,4).

Primary Health Care (PHC) systems are uniquely poised to address the problems of undernutrition and malnutrition in LMICs, especially as the world continues to face the challenges of COVID-19. PHC acts as the first point of contact between patients and the health system, allowing health care providers to identify health risks early and to deliver personalized care over a person's lifetime. PHC is delivered at the community level, therefore in a position to identify and empanel patients, provide outreach services, and continuously monitor nutrition-related indicators (5–7). Integrating essential nutrition services into routine PHC services, specifically in maternal, reproductive, newborn, and child health services (RMNCH), has the potential to greatly impact population health as good nutrition is a determinant to improved health outcomes(8).

The majority of PHC and nutrition-related research has focused on the availability of health system inputs (e.g., availability of drugs, equipment, workforce, and information systems at the facility). However, the extent to which nutrition services are integrated into routine PHC in LMICs, along with the quality of these services, is not well known. Specifically, there is a significant gap in understanding patients' experience in receiving the recommended components of high-quality care and whether nutrition services in LMICs are delivered by competent providers (9,10).

Health facility surveys such as the Service Delivery Indicators (SDI), Service Availability and Readiness Assessment (SARA) and the Service Provision Assessment (SPA) do collect information pertaining to the quality of nutrition services provided in many LMICs (11). The latter can be used to better understand key aspects of the service delivery process from both the patients' and providers' sides. This is evidenced by King et al.(11), who proposed a standardized set of measures/indices for nutrition service quality among pregnant women and children based on expert review and data available in standardized health facility surveys such as SARA and SPA.

Given that the focus of the existing literature has been on the availability of nutrition services through system inputs (9,12,13), this study bridges the gap by focusing on the service delivery processes that underpin the quality of nutrition services at the PHC level. Precisely, we assess the competence of PHC providers in delivering essential and preventive nutrition services during Antenatal care (ANC) and sick child visits and patients' experience in receiving the recommended components of care.

For peer review only - http://bmjopen.bmj.com/site/about/guidelines.xhtml

Methods

Data Sources

In this observational cross-sectional study, we utilized the Service Provision Assessment (SPA) survey for information on nutrition services provided during PHC visits. The SPA survey is a health facility assessment that provides a comprehensive overview of a country's health service delivery (14). It provides insight into the availability of various health services, service readiness to provide these services, quality of care (the extent to which the service delivery process follows generally accepted standards of care), and client satisfaction with the services offered (14). A total of 12 countries completed a SPA survey from 2007 to 2019, 11 of which had sufficient data for the present analysis. The final analytic dataset included anonymized data from the following SPA surveys: Afghanistan (2018), Democratic Republic of Congo (2018), Haiti (2017), Kenya (2010), Malawi (2013-2014), Namibia (2009), Nepal (2015), Rwanda (2007), Senegal (2018), Tanzania (2015), and Uganda (2007). Bangladesh was excluded due to the limited availability of information on several tracer items necessary for the analysis.

Metrics

For the purpose of this study, we defined PHC as services provided in outpatient consultations in public and private non-hospital SPA facilities. We specifically focused on two critical processes for high-quality service delivery: patients' experience and provider competence. For each critical process, tracer items were identified based on the set quantitative measures/tracer items of nutrition service quality for pregnant women and children proposed by King et al (11), as well as SPA data availability [see Table 1]. Patients' experience was assessed as the self-reported awareness of the nutrition services received in PHC and provider effectiveness in delivering these services. As such, patient education and support through counselling were a key focus. The client exit interview, a SPA tool in which clients provide their perceptions on the visit, was used to collect information on the patient's experience of the services offered. Eleven tracer items for patient experience were extracted from the SPA datasets across two service areas: ANC and sick child consultations. Similarly, provider competence was assessed as the direct observations of actions performed during ANC and sick child visits. Seventeen tracer activities for provider competence were extracted from SPA datasets. The observation checklist was the main SPA tool used to collect information on the identified tracer activities. Table 1 provides a breakdown of SPA tracer activities/indicators and SPA tools utilized in the present analysis.

Data Analysis

To assess patients' experience and competence of providers in delivering nutrition services during PHC visits, each of the extracted relevant tracer items was recorded to binary variables. A value of 1 indicated that a nutrition-related quality indicator, i.e., supply, counseling or advice, procedure, or training, was available or was provided, and a value of 0 indicates that the indicator was not available or provided. We then applied the recommended SPA visit, provider, and facility weights to estimate the average frequency of each of the extracted tracer indicators per country. The only exception was in Afghanistan, where data collection was limited to the urban provinces, so national averages are not representative of the entire country. A composite score was then computed for each of the two examined quality subdomains as a simple unweighted average of the 11 tracer items for patients' experience and the 17 tracer items for providers' competence. Only in Namibia and Rwanda, providers' competence subdomain score was based on 15 tracer items, and in Uganda, estimation was based on 14 tracer items due to missing data on the remainder of the tracer items. Stata 17 and R were used to analyze data and generate figures.

Patient and public involvement

This study relied on secondary analysis of publicly available datasets. Therefore, patients and the public were not involved in the formulation of this research.

For peer review only - http://bmjopen.bmj.com/site/about/guidelines.xhtml

Table 1 : List of patients' experience and provider competence tracer activities

Quality Subdomain	Thematic area	Tracer activities	SPA tool
Patients' experience	Maternal	1. Client knows at least one side effect of iron<br>2. Provider encouraged questions<br>3. Provider advised 6 months exclusive breastfeeding<br>4. Provider ever advised breastfeeding<br>5. Provider explained iron side effects in any ANC visit<br>6. Provider explained how to take iron/folate in any ANC visit	Client exit interview - Antenatal care
	Child	1. Provider asked about child's usual eating habits<br>2. Child was weighed at the facility (exit)<br>3. Provider counseled on appropriate fluid/breastfeeding<br>4. Provider counseled on appropriate solid food intake<br>5. Provider discussed child's weight and growth	Client exit interview-Curative childcare and growth monitoring services
Provider competence	Maternal	1. Provider advised exclusive breastfeeding<br>2. Provider advised early/immediate breastfeeding<br>3. Provider described how to take iron pills<br>4. Provider mentioned side effects of iron<br>5. Provider explained purpose of iron pills<br>6. Provider discussed nutrition during pregnancy<br>7. Provider checked pallor of conjunctiva/palms for pallor<br>8. Provider measured weight during ANC	Observation checklist- Antenatal care
	Child	1. Provider counselled on child weight / growth chart<br>2. Provider weighed child<br>3. Provider plotted weight on growth chart<br>4. Provider checked palms / conjunctiva / mouth for pallor<br>5. Provider asked / caretaker mentioned if child unable to drink or breastfeed<br>6. Provider asked about normal (breast)feeding pattern<br>7. Provider asked about (breast)feeding pattern during this illness<br>8. Provider asked if child received any deworming<br>9. Provider asked if child received vitamin A within 6 months	Observation checklist- Curative child care and growth monitoring services

Results

The present study included information on the quality of nutrition services in 18644 ANC visits and 23262 sick child visits in 8458 facilities across 130 subnational areas in 11 LMICS. More than half of the analyzed facilities (59 %, n=4990) were public, 76 % (n =6248) were classified as primary/non-hospital, and 39% (n=3298) of the facilities with available data were located in rural areas (Table 2). All the studied countries, except DRC, scored below 50 % in both patients' experience and provider competence (Figure 1). For example, based on the 11 tracer activities assessed for patient experience, 37% of the interviewed clients reported being aware of the nutrition services received in PHC and confirmed provider effectiveness in delivering these services. Among the 11 countries assessed, analyzed facilities in Congo scored highest in patients' experience with an overall score of 54 %, while Nepal scored lowest at 29.7%. Similarly, only about 31 % of the observed providers successfully and effectively delivered essential nutrition services in PHC based on the 17 tracer activities assessed for provider competence. Among the 11 countries assessed, Namibia scored highest in provider competence, with an overall score of 48%, while Nepal scored lowest with an average score of 20 %.

For peer review only - http://bmjopen.bmj.com/site/about/guidelines.xhtml

For peer review only

Table 2: Descriptive characteristics of the analyzed sample

	Primary* (%)	Public (%)	Rural (%)	Facilities (n)	Districts (n)	Regions (n)
Afghanistan	38	23	1	160	-	7
DRC	54	61	76	1,412	502	26
Haiti	87	34	62	1,033	34	10
Kenya	64	50	-	695	142	8
Malawi	89	48	68	1,060	28	3
Namibia	89	74	-	411	4	13
Nepal	73	78	-	992	75	5
Rwanda	92	57	-	538	-	5
Senegal	92	81	21	466	-	14
Tanzania	78	67	63	1,200	10	30
Uganda	76	71	-	491	-	9
**Total**	**76**	**59**	**39**	**8,458**	**1297**	**130**

*Primary level was defined as services provided in outpatient consultations in public and private non-hospital SPA facilities

Figure 1: Quality of nutrition services by subdomain score in each of the studied countries

Assessment of individual tracer items of patients' experience with maternal nutrition services revealed that more than half of clients were encouraged to ask questions during ANC visits in 9 out of the 11 studied countries. In addition, more than 80 % of clients in each of the 11 studied countries reported being advised on taking iron supplements during pregnancy. However, less than 40 % of patients reported being advised on iron side effects. In contrast, when asked in an exit interview, more than 50 % of clients knew at least one side effect of iron in 6 out of the 11 countries assessed. In addition, less than 40 % of clients reported being advised on exclusive breastfeeding in each of the studied countries, and only in Malawi and Namibia, more than 50 % of clients were ever advised on breastfeeding during ANC (Table 3).

Patients' experience with child nutrition services was found to be significantly lagging compared to maternal services. In five out of the 11 countries analyzed, more than 70 % of clients reported weighing the child at the facility; however, less than half of clients reported that the child's weight and growth were discussed. Moreover, in 9 out of the 11 countries analyzed, less than 25 % of clients reported being asked about the child's eating habits. In addition, in the majority of studied countries (10 out of 11), at most, 41 % of clients were counseled on appropriate fluid intake or breastfeeding, and less than 37 % were counseled on appropriate solid food intake. The only exception was DRC, where almost all clients (99%) reported being advised on both appropriate fluid and appropriate solid food intake (Table 3).

Table 3: Patient experience with maternal and child nutrition services

Patients' experience tracer items		AF	DRC	HT	KE	MW	NM	NP	RW	SN	TZ	UG
Maternal	Client knows at least one side effect of iron	55%	52%	71%	42%	75%	30%	20%	9%	72%	59%	22%
	Provider explained iron side effects in any ANC visit	29%	22%	16%	23%	17%	34%	24%	13%	37%	29%	17%
	Provider explained how to take iron/folate in any ANC visit	83%	87%	85%	95%	96%	96%	95%	88%	93%	95%	87%
	Provider encouraged questions	29%	51%	61%	63%	80%	65%	37%	57%	66%	78%	62%

For peer review only - http://bmjopen.bmj.com/site/about/guidelines.xhtml

	Provider advised 6 months exclusive breastfeeding	6%	20%	16%	28%	36%	13%	14%	28%	11%	29%	19%
	Provider ever advised breastfeeding	12%	27%	30%	35%	52%	65%	23%	32%	16%	38%	30%
Child	Provider asked about child's usual eating habits	32%	23%	25%	20%	13%	0%	14%	10%	39%	21%	19%
	Child was weighed at the facility (exit)	47%	78%	89%	46%	33%	87%	58%	72%	93%	30%	51%
	Provider counseled on appropriate fluid/breastfeeding	39%	100 %	25%	18%	12%	28%	29%	13%	33%	18%	41%
	Provider counseled on appropriate solid food intake	36%	99%	14%	15%	9%	25%	21%	12%	40%	11%	26%
	Provider discussed child's weight and growth	31%	36%	35%	23%	17%	35%	18%	45%	40%	35%	21%

Assessment of tracer activities for providers' competence in maternal nutrition services revealed that weight was measured during the ANC consultation for the majority (more than 70 %) of clients in 10 out of the 11 analyzed countries. The only exception was in Afghanistan, where only 26 % of observed providers measured the mother's weight. Signs of anemia were also checked by more than half of providers in 8 out of the ten countries with available data. However, in 7 out of the 11 countries analyzed, less than half of observed providers explained the purpose of iron or how to take iron pills. Furthermore, less than 15 % of providers mentioned the side effects of iron in all the studied countries. Counseling on breastfeeding was also one of the areas with significant gaps. Specifically, less than 20 % of observed providers advised on early/ immediate breastfeeding in all countries with available data (8 countries), and less than 40 % of observed providers advised exclusive breastfeeding in all the 11 studied countries (Table 4).

Table 4: Providers' competence in maternal and child nutrition services

Provider competence tracer items		AF	DRC	HT	KE	MW	NM	NP	RW	SN	TZ	UG
Maternal	Provider advised exclusive breastfeeding	7%	8%	6%	22%	6%	37%	1%	19%	6%	11%	15%
	Provider advised early/immediate breastfeeding	6%	5%	3%	18%	4%	-	2%	-	4%	7%	-
	Provider described how to take iron pills	38%	35%	38%	46%	57%	64%	22%	35%	56%	56%	46%
	Provider mentioned side effects of iron	11%	5%	4%	12%	8%	13%	4%	14%	7%	9%	6%
	Provider explained purpose of iron pills	27%	28%	33%	41%	59%	52%	22%	31%	54%	56%	36%
	Provider discussed nutrition during pregnancy	54%	29%	51%	49%	36%	48%	53%	39%	50%	35%	38%
	Provider checked pallor of conjunctiva/palms for pallor	36%	56%	51%	78%	79%	59%	36%	78%	86%	54%	-
	Provider measured weight during ANC	26%	74%	89%	96%	79%	93%	77%	99%	94%	78%	79%
Child	Provider counselled on child weight / growth chart	15%	10%	8%	26%	10%	33%	6%	17%	17%	25%	10%
	Provider weighed child	45%	74%	87%	45%	20%	87%	47%	67%	93%	15%	50%
	Provider plotted weight on growth chart	4%	7%	9%	21%	6%	51%	19%	9%	56%	4%	13%
	Provider checked palms / conjunctiva / mouth for pallor	27%	75%	48%	56%	48%	41%	15%	37%	24%	38%	74%
	Provider asked / caretaker mentioned if child unable to drink or breastfeed	20%	23%	19%	44%	29%	45%	25%	27%	3%	28%	62%

For peer review only - http://bmjopen.bmj.com/site/about/guidelines.xhtml

	Provider asked about normal (breast)feeding pattern	25%	32%	36%	42%	16%	47%	18%	32%	30%	27%	44%
	Provider asked about (breast)feeding pattern during this illness	19%	28%	28%	47%	17%	47%	18%	46%	7%	20%	42%
	Provider asked if child received any deworming	1%	11%	3%	21%	2%	-	3%	-	28%	5%	-
	Provider asked if child received vitamin A within 6 months	4%	6%	2%	20%	2%	34%	1%	16%	31%	2%	12%

Note: -No data is available

Similarly, measuring weight or checking for signs of anemia during sick child consultations were among the highest-scored tracer activities of provider competence. However, less than one-third (33%) of observed providers counseled clients on child growth in each of the studied countries and less than one quarter (21%) plotted the child weight on a growth chart in 9 out of the 11 countries studied. In addition, less than 50 % of observed providers in each of the analyzed countries asked about the child's normal feeding pattern or feeding pattern during illness. Furthermore, in 8 out of the 11 countries analyzed, less than 30 % of observed providers asked whether the child was unable to feed or drink during sick child consultations. Also, whenever data was available, 34 % of providers, at most, asked if the child received vitamin A or a deworming medication in each of the studied countries (Table 4).

Discussion

Overall, the descriptive analysis of patients' experience and provider competence in the present study showed the delivery of low-quality maternal and child nutrition services at the PHC level across 11 LMICs, where only one country (DRC) reported a provider competence score above 50%. These findings highlight the urgent need to integrate quality essential and preventive nutrition services into routine PHC in LMICs.

We also found compelling evidence indicating the lack of depth in providers' assessment of the nutritional status of expectant mothers and children. Across all assessed countries, providers commonly took anthropometric measurements of expectant mothers and children; however, such assessments were not always followed up with advice or counseling about growth patterns, developmental milestones, or dietary advice which is critically important for mitigating both undernutrition and overweight. The WHO child growth standard also recommends that children's weight and height are plotted on the standard growth chart following a physical examination, as they can be good predictors of dietary status and future disease risk when followed over time (15).

Explaining side effects of iron by providers was also one of the most sub-optimally performed activities from both the patient and the provider perspectives. Iron supplements are required to be taken daily, and empirical research shows that taking iron supplements is often accompanied by side effects such as constipation, cramps, etc., making treatment adherence difficult(16). It is important that providers are trained to forewarn anemic pregnant women of both the benefits and side-effects to attain treatment compliance and better health outcomes(16,17). Failure to counsel women on the proximal and long-lasting maternal, neonatal, and child health benefits of iron supplementation and breastfeeding during antenatal care represents a missed opportunity for improving maternal and child health(18). It is also important to consider that from a measurement perspective, many nutrition services and campaigns can be based in the community, which might explain the notably better performance in the patients' knowledge and awareness of Iron side effects, incongruent to the levels of provider competence in this analysis. Since the SPA data utilized in the present analysis only focuses on facility-based nutrition services, future research and tools should consider capturing the provision of community-based nutrition services.

For peer review only

For peer review only - http://bmjopen.bmj.com/site/about/guidelines.xhtml

This analysis also suggests that patient health literacy and confidence may be a determinant of receiving high-quality nutrition services. Positive patient perception of patient-provider interactions and quality of care is associated with better health experiences (19). Over 70 percent of women in two out of 11 countries reviewed showed that there was a lack of providers who encouraged women to ask questions during ANC. Positive provider interaction with mothers in the healthcare environment has been shown to be psychodynamic and therapeutic, thereby leading to greater patient satisfaction with the quality of care (20).To encourage question-asking behavior, it is important that health providers use simple and empathic language when dealing with expectant mothers and allow them to express themselves without fear (21).

Comprehensive SPA surveys continue to be essential tools in the assessment of health service delivery in LMICs. However, the surveys lack an array of vital evidence-based nutrition interventions such as calcium, balanced energy, and protein, multiple micronutrient supplementation in pregnancy. The current versions of the survey also do not adequately capture treatment of uncomplicated Severe Acute Malnutrition (SAM) through multiple micronutrient powder and supplements and small quantity lipid-based nutrient supplements (SQ-LNS) in children (22–25). Further, facility surveys generally lack specificity in topics key to nutrition assessment and training, especially in non-communicable disease services.

Notably, our findings give rise to additional questions in understanding and framing the drivers influencing the provision of quality nutrition services in PHC. While the availability of nutrition services through investments in system inputs (drugs, diagnostics, equipment, workforce, and information systems) is a cornerstone for quality of care, service delivery processes such as having competent providers and clients who are engaged in their care is key to achieving the effective coverage for nutrition services (26,27). Future research should also consider the use of available service delivery indicators to understand the relationship between the quality of nutrition service delivery and nutrition-related health outcomes in LMICS.

Conclusion

Although provider competence and patients 'experience across the 11 assessed countries demonstrated the basic delivery of nutrition services, the apparent deficiency in the extent and depth of questions asked for the majority of assessed tracer activities reveal significant opportunities for improving the quality of nutrition service delivery process at the PHC level. Measuring the quality of nutrition services in PHC also remains a challenge due to the limited nature of some of the existing indicators; however, multiple opportunities exist for improving and utilizing the current tools.

Ethics statements

Data included in the present analysis were extracted from anonymized publicly available datasets and no patient consent was needed

Availability of data and material

The original datasets analyzed during the current study are available from the DHS program website, https://dhsprogram.com/data/available-datasets.cfm. The analytical dataset generated during the current study is available from the corresponding author on reasonable request

Authors contributions : MR,TBM and MVU were responsible for the conception of this study and for methods development. MR and TBM analysed data. MR, TBM, LO and CF prepared the first draft. MR, LO, CF, MVU all contributed to subsequent iterations of the draft. All authors reviewed results or reviewed and contributed to the final manuscript.

For peer review only - http://bmjopen.bmj.com/site/about/guidelines.xhtml

Funding: This research was supported, in whole or in part, by the Bill and Melinda Gates Foundation (Grant number INV000932), and it had no role in study design, data collection, data analysis, data interpretation or writing of the manuscript.

Competing interests: None declared.

For peer review only

References

1. UNICEF. 2020 Global Nutrition Report - Global Nutrition Report [Internet]. 2020 [cited 2020 Nov 2]. Available from: https://globalnutritionreport.org/reports/2020-global-nutrition-report/

2. Murray CJL, Aravkin AY, Zheng P, Abbafati C, Abbas KM, Abbasi-Kangevari M, et al. Global burden of 87 risk factors in 204 countries and territories, 1990–2019: a systematic analysis for the Global Burden of Disease Study 2019. The Lancet. 2020 Oct 17;396(10258):1223–49.

3. United Nations. Policy Brief: The Impact of COVID-19 on Food Security and Nutrition |Policy Support and Governance| Food and Agriculture Organization of the United Nations [Internet]. Un. 2020 [cited 2021 Apr 17]. Available from: http://www.fao.org/policy-support/tools-and-publications/resources-details/en/c/1287907/

4. World Bank. Food Security and COVID-19 [Internet]. Brief. 2021 [cited 2021 Apr 17]. Available from: https://www.worldbank.org/en/topic/agriculture/brief/food-security-and-covid-19

5. Kraef C, Wood B, Von Philipsborn P, Singh S, Peterson SS. Primary health care and nutrition. 2020.

6. America I of MC on Q of HC in. Crossing the Quality Chasm: A New Health System for the 21st Century. J Healthc Qual. 2002;24(5):52.

7. Primary Health Care Performance Initiative. The PHCPI Conceptual Framework [Internet]. 2018 [cited 2020 Nov 28]. Available from: https://improvingphc.org/phcpi-conceptual-framework

8. Nutrition [Internet]. [cited 2022 Mar 26]. Available from: https://www.who.int/news-room/facts-in-pictures/detail/nutrition

9. Amouzou A, Leslie HH, Ram M, Fox M, Jiwani SS, Requejo J, et al. Advances in the measurement of coverage for RMNCH and nutrition: from contact to effective coverage. BMJ Glob Health. 2019 May 1;4(Suppl 4):e001297.

10. Gillespie S, Menon P, Heidkamp R, Piwoz E, Rawat R, Munos M, et al. Measuring the coverage of nutrition interventions along the continuum of care: time to act at scale. BMJ Glob Health. 2019;4(Suppl 4):e001290.

11. King SE, Sheffel A, Heidkamp R, Xu YY, Walton S, Munos MK. Advancing nutrition measurement: Developing quantitative measures of nutrition service quality for pregnant women and children in low- and middle-income country health systems. Matern Child Nutr. 2022 Jan;18(1):e13279.

12. Mallick L, Temsah G, Benedict RK. Facility-based nutrition readiness and delivery of maternal and child nutrition services using service provision assessment surveys. 2018 Sep 1 [cited 2022 Mar 26]; Available from: https://dhsprogram.com/publications/publication-cr49-comparative-reports.cfm

13. Marsh AD, Muzigaba M, Diaz T, Requejo J, Jackson D, Chou D, et al. Effective coverage measurement in maternal, newborn, child, and adolescent health and nutrition: progress, future prospects, and implications for quality health systems. Lancet Glob Health. 2020 May;8(5):e730–6.

14. USAID. The DHS Program - Service Provision Assessments (SPA) [Internet]. Demographic Health Surveys. 2019 [cited 2020 Nov 28]. p. 1–2. Available from: https://dhsprogram.com/What-We-Do/Survey-Types/SPA.cfm

For peer review only - http://bmjopen.bmj.com/site/about/guidelines.xhtml

15. Child growth standards [Internet]. [cited 2022 Mar 26]. Available from: https://www.who.int/tools/child-growth-standards

16. Christian P, Mullany LC, Hurley KM, Katz J, Black RE. Nutrition and maternal, neonatal, and child health. Semin Perinatol. 2015 Aug;39(5):361–72.

17. Smith ER, Shankar AH, Wu LSF, Aboud S, Adu-Afarwuah S, Ali H, et al. Modifiers of the effect of maternal multiple micronutrient supplementation on stillbirth, birth outcomes, and infant mortality: a meta-analysis of individual patient data from 17 randomised trials in low-income and middle-income countries. Lancet Glob Health. 2017 Nov;5(11):e1090–100.

18. Joseph NT, Piwoz E, Lee D, Malata A, Leslie HH. Examining coverage, content, and impact of maternal nutrition interventions: the case for quality-adjusted coverage measurement. J Glob Health. 10(1):010501.

19. Ardey R, Ardey R. Patient Perceptions and Expectations From Primary Health-care Providers in India. J Fam Med Prim Care. 2015 Mar;4(1):53–63.

20. Perla L. Patient compliance and satisfaction with nursing care during delivery and recovery. J Nurs Care Qual. 2002 Jan;16(2):60–6.

21. Redshaw M. Women as consumers of maternity care: measuring “satisfaction” or “dissatisfaction”? Birth Berkeley Calif. 2008 Mar;35(1):73–6.

22. WHO. Calcium supplementation during pregnancy to reduce the risk of pre-eclampsia. Who. 2017;

23. Smuts CM, Matsungo TM, Malan L, Kruger HS, Rothman M, Kvalsvig JD, et al. Effect of small-quantity lipid-based nutrient supplements on growth, psychomotor development, iron status, and morbidity among 6-to 12-mo-old infants in South Africa: A randomized controlled trial. Am J Clin Nutr. 2019 Jan 1;109(1):55–68.

24. Stevens B, Buettner P, Watt K, Clough A, Brimblecombe J, Judd J. The effect of balanced protein energy supplementation in undernourished pregnant women and child physical growth in low- and middle-income countries: A systematic review and meta-analysis. Vol. 11, Maternal and Child Nutrition. Blackwell Publishing Ltd; 2015. p. 415–32.

25. Huybregts L, Houngbé F, Salpéteur C, Brown R, Roberfroid D, Ait-Aissa M, et al. The Effect of Adding Ready-to Use Supplementary Food to a General Food Distribution on Child Nutritional Status and Morbidity: A Cluster-Randomized Controlled Trial. Prentice A, editor. PLoS Med. 2012 Sep 18;9(9):e1001313.

26. Amouzou A, Leslie HH, Ram M, Fox M, Jiwani SS, Requejo J, et al. Advances in the measurement of coverage for RMNCH and nutrition: from contact to effective coverage. BMJ Glob Health. 2019;4:114–24.

27. Nguyen PH, Khuong LQ, Pramanik P, Billah SM, Menon P, Piwoz E, et al. Effective coverage of nutrition interventions across the continuum of care in Bangladesh: Insights from nationwide cross-sectional household and health facility surveys. BMJ Open. 2021 Jan 20;11(1):40109.

For peer review only - http://bmjopen.bmj.com/site/about/guidelines.xhtml

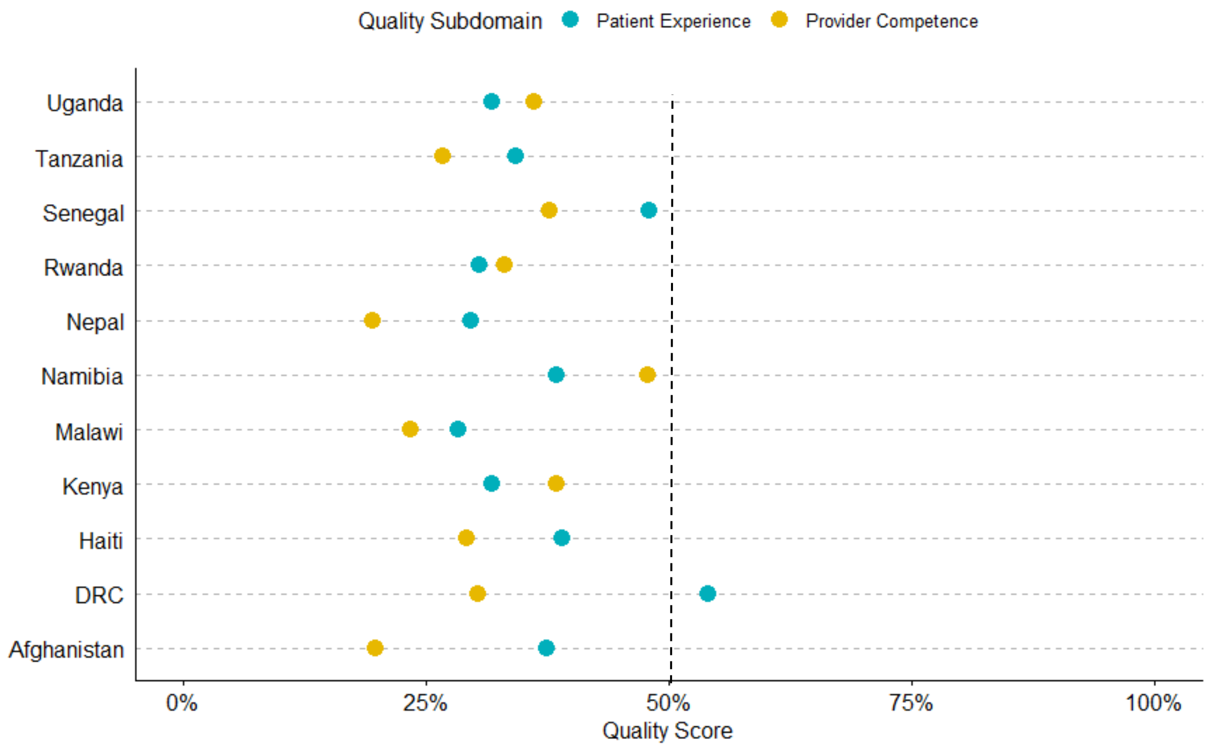

For peer review only - http://bmjopen.bmj.com/site/about/guidelines.xhtml

The RECORD statement – checklist of items, extended from the STROBE statement, that should be reported in observational studies using routinely collected health data.

	Item No.	STROBE items	Location in manuscript where items are reported	RECORD items	Location in manuscript where items are reported
**Title and abstract**					
	1	(a) Indicate the study's design with a commonly used term in the title or the abstract (b) Provide in the abstract an informative and balanced summary of what was done and what was found	(a) line 21, page 2<br><br>(b) lines 19-39, page 2	RECORD 1.1: The type of data used should be specified in the title or abstract. When possible, the name of the databases used should be included.<br><br>RECORD 1.2: If applicable, the geographic region and timeframe within which the study took place should be reported in the title or abstract.<br><br>RECORD 1.3: If linkage between databases was conducted for the study, this should be clearly stated in the title or abstract.	1.1: line 22, page 2<br><br>1.2: lines 22-25, page 2<br><br>1.3: NA
**Introduction**					
Background rationale	2	Explain the scientific background and rationale for the investigation being reported	Lines 53-83, page 3		
Objectives	3	State specific objectives, including any prespecified hypotheses	Lines 84-88, page 3		
**Methods**					
Study Design	4	Present key elements of study design early in the paper	Lines 92, page 4		
Setting	5	Describe the setting, locations, and relevant dates, including periods of recruitment, exposure, follow-up, and data collection	Lines 92-101, page 4		

For peer review only

Participants	6	*(a) Cohort study* - Give the eligibility criteria, and the sources and methods of selection of participants. Describe methods of follow-up<br>*Case-control study* - Give the eligibility criteria, and the sources and methods of case ascertainment and control selection. Give the rationale for the choice of cases and controls<br>*Cross-sectional study* - Give the eligibility criteria, and the sources and methods of selection of participants<br><br>*(b) Cohort study* - For matched studies, give matching criteria and number of exposed and unexposed<br>*Case-control study* - For matched studies, give matching criteria and the number of controls per case	NA	RECORD 6.1: The methods of study population selection (such as codes or algorithms used to identify subjects) should be listed in detail. If this is not possible, an explanation should be provided.<br><br>RECORD 6.2: Any validation studies of the codes or algorithms used to select the population should be referenced. If validation was conducted for this study and not published elsewhere, detailed methods and results should be provided.<br><br>RECORD 6.3: If the study involved linkage of databases, consider use of a flow diagram or other graphical display to demonstrate the data linkage process, including the number of individuals with linked data at each stage.	6.1: NA- secondary analysis of publicly available anonymized data was used<br>6.2: NA- secondary analysis of publicly available anonymized data was used<br><br>6.3: NA- no linkage was done
Variables	7	Clearly define all outcomes, exposures, predictors, potential confounders, and effect modifiers. Give diagnostic criteria, if applicable.	Lines 103-116, page 5<br>Table 1-Page 6	RECORD 7.1: A complete list of codes and algorithms used to classify exposures, outcomes, confounders, and effect modifiers should be provided. If these cannot be reported, an explanation should be provided.	Lines 103-116, page 5<br>Table 1-Page 6<br><br>Confounders and effect modifiers were not measures in descriptive analysis – no regression models were carried out t
Data sources/ measurement	8	For each variable of interest, give sources of data and details	Lines 92-101<br>Table 1 -page 6		

For peer review only - http://bmjopen.bmj.com/site/about/guidelines.xhtml

		of methods of assessment (measurement). Describe comparability of assessment methods if there is more than one group			
Bias	9	Describe any efforts to address potential sources of bias	NA		
Study size	10	Explain how the study size was arrived at	Lines 92-101, page 5		
Quantitative variables	11	Explain how quantitative variables were handled in the analyses. If applicable, describe which groupings were chosen, and why	Lines 102-129, page 5		
Statistical methods	12	(a) Describe all statistical methods, including those used to control for confounding<br>(b) Describe any methods used to examine subgroups and interactions<br>(c) Explain how missing data were addressed<br>(d) *Cohort study* - If applicable, explain how loss to follow-up was addressed<br>*Case-control study* - If applicable, explain how matching of cases and controls was addressed<br>*Cross-sectional study* - If applicable, describe analytical methods taking account of sampling strategy<br>(e) Describe any sensitivity analyses	a) Lines 118-129, page 5<br>b) NA<br>c)Lines 126-129, page 5<br>d)NA<br>e)NA		

For peer review only

Data access and cleaning methods		..		RECORD 12.1: Authors should describe the extent to which the investigators had access to the database population used to create the study population.<br><br>RECORD 12.2: Authors should provide information on the data cleaning methods used in the study.	12.1: Data is publicly available (line 131, page 5) so section 12.1 does not apply<br><br>12.2: NA as access to already cleaned data was provided through DHS website
Linkage		..		RECORD 12.3: State whether the study included person-level, institutional-level, or other data linkage across two or more databases. The methods of linkage and methods of linkage quality evaluation should be provided.	12.3: NA
**Results**					
Participants	13	(a) Report the numbers of individuals at each stage of the study (*e.g.*, numbers potentially eligible, examined for eligibility, confirmed eligible, included in the study, completing follow-up, and analysed)<br>(b) Give reasons for non-participation at each stage.<br>(c) Consider use of a flow diagram	(a), (b),(c) are not applicable as the study is based on secondary data analysis	RECORD 13.1: Describe in detail the selection of the persons included in the study (*i.e.,* study population selection) including filtering based on data quality, data availability and linkage. The selection of included persons can be described in the text and/or by means of the study flow diagram.	13.1: lines 135-136, page 6
Descriptive data	14	(a) Give characteristics of study participants (*e.g.*, demographic, clinical, social) and information on exposures and potential confounders	Demographic of patients is not applicable; characteristics of population are described in lines		

For peer review only - http://bmjopen.bmj.com/site/about/guidelines.xhtml

		(b) Indicate the number of participants with missing data for each variable of interest<br>(c) *Cohort study* - summarise follow-up time (*e.g.*, average and total amount)	135-139, page 6 and Table 2, page 7		
Outcome data	15	*Cohort study* - Report numbers of outcome events or summary measures over time<br>*Case-control study* - Report numbers in each exposure category, or summary measures of exposure<br>*Cross-sectional study* - Report numbers of outcome events or summary measures	Lines 151-155, page 6		
Main results	16	(a) Give unadjusted estimates and, if applicable, confounder-adjusted estimates and their precision (e.g., 95% confidence interval). Make clear which confounders were adjusted for and why they were included<br>(b) Report category boundaries when continuous variables were categorized<br>(c) If relevant, consider translating estimates of relative risk into absolute risk for a meaningful time period	(a) Tables3-page 7 & Table 4-page 8<br><br>(b) NA<br><br>(c) NA		
Other analyses	17	Report other analyses done—e.g., analyses of subgroups and interactions, and sensitivity analyses	NA		
**Discussion**					
Key results	18	Summarise key results with reference to study objectives	Line 190-193, page 9		

For peer review only

Limitations	19	Discuss limitations of the study, taking into account sources of potential bias or imprecision. Discuss both direction and magnitude of any potential bias	Line 208-212, page 9<br>Lines 220-227, page 10	RECORD 19.1: Discuss the implications of using data that were not created or collected to answer the specific research question(s). Include discussion of misclassification bias, unmeasured confounding, missing data, and changing eligibility over time, as they pertain to the study being reported.	Line 208-212, page 9<br>Lines 220-227, page 10
Interpretation	20	Give a cautious overall interpretation of results considering objectives, limitations, multiplicity of analyses, results from similar studies, and other relevant evidence	Line 194-220, page 9-10		
Generalisability	21	Discuss the generalisability (external validity) of the study results	NA as all countries conducted a SPA survey were included in the present study		
**Other Information**					
Funding	22	Give the source of funding and the role of the funders for the present study and, if applicable, for the original study on which the present article is based	Lines 253-255, page 11		
Accessibility of protocol, raw data, and programming code		..		RECORD 22.1: Authors should provide information on how to access any supplemental information such as the study protocol, raw data, or programming code.	Lines 245-248, page 10

*Reference: Benchimol EI, Smeeth L, Guttmann A, Harron K, Moher D, Petersen I, Sørensen HT, von Elm E, Langan SM, the RECORD Working Committee. The REporting of studies Conducted using Observational Routinely-collected health Data (RECORD) Statement. *PLoS Medicine* 2015; in press.

*Checklist is protected under Creative Commons Attribution (CC BY) license.

BMJ Open

Existing gaps and missed opportunities in delivering quality nutrition services in primary healthcare: a descriptive analysis of patient experience and provider competence in 11 low and middle-income countries

Journal:	*BMJ Open*
Manuscript ID	bmjopen-2022-064819.R1
Article Type:	Original research
Date Submitted by the Author:	14-Dec-2022
Complete List of Authors:	Ramadan, Marwa; World Bank Group<br>Muthee, Tonny B; World Bank Group<br>Okara, Latifat; World Bank Group<br>Feil, Cameron; World Bank Group<br>Villar-Uribe , Manuela; World Bank Group
**Primary Subject Heading**:	Nutrition and metabolism
Secondary Subject Heading:	Global health, Public health
Keywords:	Nutrition < TROPICAL MEDICINE, PUBLIC HEALTH, PRIMARY CARE, Quality in health care < HEALTH SERVICES ADMINISTRATION & MANAGEMENT

SCHOLARONE™
Manuscripts

I, the Submitting Author has the right to grant and does grant on behalf of all authors of the Work (as defined in the below author licence), an exclusive licence and/or a non-exclusive licence for contributions from authors who are: i) UK Crown employees; ii) where BMJ has agreed a CC-BY licence shall apply, and/or iii) in accordance with the terms applicable for US Federal Government officers or employees acting as part of their official duties; on a worldwide, perpetual, irrevocable, royalty-free basis to BMJ Publishing Group Ltd ("BMJ") its licensees and where the relevant Journal is co-owned by BMJ to the co-owners of the Journal, to publish the Work in this journal and any other BMJ products and to exploit all rights, as set out in our licence.

The Submitting Author accepts and understands that any supply made under these terms is made by BMJ to the Submitting Author unless you are acting as an employee on behalf of your employer or a postgraduate student of an affiliated institution which is paying any applicable article publishing charge ("APC") for Open Access articles. Where the Submitting Author wishes to make the Work available on an Open Access basis (and intends to pay the relevant APC), the terms of reuse of such Open Access shall be governed by a Creative Commons licence – details of these licences and which Creative Commons licence will apply to this Work are set out in our licence referred to above.

Other than as permitted in any relevant BMJ Author's Self Archiving Policies, I confirm this Work has not been accepted for publication elsewhere, is not being considered for publication elsewhere and does not duplicate material already published. I confirm all authors consent to publication of this Work and authorise the granting of this licence.

For peer review only

Existing gaps and missed opportunities in delivering quality nutrition services in primary healthcare: a descriptive analysis of patient experience and provider competence in 11 low and middle-income countries

Authors and affiliations

Marwa Ramadan[1]*, Tonny B Muthee[1] , Latifat Okara[1], Cameron Feil[1], Manuela Villar Uribe[1]

[1]Health Nutrition and Population Global Practice, World Bank Group, Washington, DC, USA.

***Corresponding Author**

Marwa Ramadan

Health Nutrition and Population Global Practice, World Bank Group, Washington, DC, USA

mramadan@worldbank.org

Keywords

Primary health care; nutrition services; healthcare quality; measurement; Low- and Middle-income countries

Total word count : 4934

Abstract word count: 282

Number of references : 24

For peer review only

Abstract

Objectives: To assess the competence of PHC providers in delivering maternal and child nutrition services at the PHC level and patients' experience in receiving the recommended components of care

Design: Observational cross-sectional analysis

Setting: Healthcare facilities in Low- and Middle-Income Countries (LMICs) with available service provision assessment surveys (Afghanistan (2018), Democratic Republic of Congo (2018), Haiti (2017), Kenya (2010), Malawi (2013-2014), Namibia (2009), Nepal (2015), Rwanda (2007), Senegal (2018), Tanzania (2015), and Uganda (2007).

Participants: 18644 antenatal visits and 23262 sick child visits in 8458 facilities across 130 subnational areas in 11 LMICs from 2007 to 2019.

Outcomes: 1) Provider competence assessed as the direct observations of actions performed during antenatal care and sick child visits; and 2) Patients 'experience defined as the self-reported awareness of the nutrition services received during ANC and Sick child visits and provider effectiveness in delivering these services

Results: Except for DRC, all countries scored below 50 % on patients' experience and provider competence. More than 70% of clients were advised on taking iron supplements during pregnancy; however, less than 32 % of patients were advised on iron side effects in all the studied countries. Across all countries, providers commonly took anthropometric measurements of expectant mothers and children; however, such assessments were rarely followed up with advice or counseling about growth patterns. In addition, less than 20 % of observed providers advised on early/ immediate breastfeeding in all countries with available data.

Conclusion: The 11 assessed countries demonstrated the delivery of limited nutrition services; nonetheless, the apparent deficiency in the extent and depth of questions asked for the majority of tracer activities revealed significant opportunities for improving the quality of nutrition service delivery at the PHC level.

For peer review only - http://bmjopen.bmj.com/site/about/guidelines.xhtml

Strengths and limitations

- This study utilizes standardized publicly available health facility surveys to capture both the patient and the provider perspectives in delivering quality nutrition services in 11 LMICs.
- Rather than solely focusing on the availability of nutrition services through system inputs, this study assesses the service delivery processes that underpin the quality of nutrition services at the PHC level.
- This study only examined the quality of nutrition services in antenatal care and child health visits, data and tools were inadequate for other key PHC services as non-communicable diseases.
- This study is descriptive in nature; inferential statistics were not utilized to statistically test or account for potential temporal and geographic variations across countries.
- The tools utilized in the present analysis only focused on facility-based nutrition services with inability to capture community-based nutrition services.

For peer review only

For peer review only - http://bmjopen.bmj.com/site/about/guidelines.xhtml

Background

Over the past 20 years, significant progress has been made in nutrition-related outcomes in many Low- and Middle-Income countries (LMICS). Many LMICs, however, now face the dual malnutrition burden of undernutrition and overweight, posing significant challenges for progress in nutrition, population health, and human capital development. Globally, it is estimated that one out of every nine people is hungry or undernourished and that one in every three people is overweight or obese (1,2). Furthermore, the food insecurity caused by the COVID-19 pandemic and ongoing conflicts such as in Ukraine and Russia are additional threats to nutrition-related progress. The United Nations estimates that 2.37 billion people lacked access to adequate food sources in 2020, an increase of 320 million people compared to 2019. The combined effects of the pandemic, and conflict-related turmoil if left unmitigated, will likely result in longstanding and severe consequences for health and nutrition (3,4).

Primary Health Care (PHC) systems are uniquely poised to address the problems of undernutrition and malnutrition in LMICs, especially as the world continues to face the challenges of COVID-19. PHC acts as the first point of contact between patients and the health system, allowing health care providers to identify health risks early and to deliver personalized care over a person's lifetime. PHC is delivered at the community level, therefore in a position to identify and empanel patients, provide outreach services, and continuously monitor nutrition-related indicators (5–7). Integrating essential nutrition services into routine PHC services, specifically in maternal, reproductive, newborn, and child health services (RMNCH), has the potential to greatly impact population health as good nutrition is a determinant to improved health outcomes(8).

The majority of PHC and nutrition-related research has focused on the availability of health system inputs (e.g., availability of drugs, equipment, workforce, and information systems at the facility). However, the extent to which nutrition services are integrated into routine PHC in LMICs, along with the quality of these services, is not well known. Specifically, there is a significant gap in understanding patients' experience in receiving the recommended components of high-quality care and whether nutrition services in LMICs are delivered by competent providers (9,10).

Health facility surveys such as the Service Delivery Indicators (SDI), Service Availability and Readiness Assessment (SARA) and the Service Provision Assessment (SPA) do collect information pertaining to the quality of nutrition services provided in many LMICs (11). The latter can be used to better understand key aspects of the service delivery process from both the patients' and providers' sides. This is evidenced by King et al.(11), who proposed a standardized set of measures/indices for nutrition service quality among pregnant women and children based on expert review and data available in standardized health facility surveys such as SARA and SPA.

Given that the focus of the existing literature has been on the availability of nutrition services through system inputs (9,12,13), this study bridges the gap by focusing on the service delivery processes that underpin the quality of nutrition services at the PHC level. Precisely, we assess the competence of PHC providers in delivering essential and preventive nutrition services during Antenatal care (ANC) and sick child visits and patients' experience in receiving the recommended components of care.

For peer review only

For peer review only - http://bmjopen.bmj.com/site/about/guidelines.xhtml

Methods

Data Sources

In this observational cross-sectional study, we utilized the Service Provision Assessment (SPA) survey for information on nutrition services provided during PHC visits. The SPA survey is a health facility assessment that provides a comprehensive overview of a country's health service delivery (14). It provides insight into the availability of various health services, service readiness to provide these services, quality of care (the extent to which the service delivery process follows generally accepted standards of care), and client satisfaction with the services offered (14). A total of 12 countries completed a SPA survey from 2007 to 2019, 11 of which had sufficient data for the present analysis. The final analytic dataset included anonymized data from the following SPA surveys: Afghanistan (2018), Democratic Republic of Congo (2018), Haiti (2017), Kenya (2010), Malawi (2013-2014), Namibia (2009), Nepal (2015), Rwanda (2007), Senegal (2018), Tanzania (2015), and Uganda (2007). Bangladesh was excluded due to the limited availability of information on several tracer items necessary for the analysis.

Metrics

In this study, we defined PHC as services provided in outpatient consultations in public and private non-hospital SPA facilities. We specifically focused on two critical processes for high-quality service delivery: patients' experience and provider competence. For each critical process, tracer items were identified based on the set quantitative measures/tracer items of nutrition service quality for pregnant women and children proposed by King et al (11), the list of SPA tracer items for measuring facility service delivery of nutrition services (12), as well as SPA data availability across the studied countries [see Table 1]. Patients' experience was assessed as the self-reported awareness of the nutrition services received in PHC and provider effectiveness in delivering these services. As such, patient education and support through counselling were a key focus. The client exit interview, a SPA tool in which clients provide their perceptions on the visit, was used to collect information on the patient's experience of the services offered. Twelve tracer items for patient experience were extracted from the SPA datasets across two service areas: ANC and sick child consultations. Similarly, provider competence was assessed as the direct observations of actions performed during ANC and sick child visits. Seventeen tracer activities for provider competence were extracted from SPA datasets. The observation checklist was the main SPA tool used to collect information on the identified tracer activities. Table 1 provides a breakdown of SPA tracer activities/indicators and SPA tools utilized in the present analysis. Detailed information on each of the utilized SPA tools are provided by the demographic and health surveys program and publicly available on its website (15).

Data Analysis

To assess patients' experience and competence of providers in delivering nutrition services during PHC visits, each of the extracted relevant tracer items (Table 1) was recoded to a binary (0,1) variable/indicator, where a value of 1 indicated that the tracer item was available or provided, and a value of 0 indicates that the tracer item was not available or provided. Next, we calculated the facility-level averages for each of the selected tracer items. Given that SPA surveys are hierarchal in nature where country data is collected at three levels(the facility, the provider, and the client), we applied the country-specific survey weights for all levels to ensure that national averages were representative per country. The only exception was in Afghanistan, where SPA data collection was limited to the urban provinces, so national averages were not representative of the entire primary health care facilities in the country. We reported national estimates for individual tracer items per country. In addition, we calculated two summary scores for each of patient experience and provider competence sub-domains. Summary scores were calculated as simple unweighted averages of the 12 tracer items for patients' experience and the 17 tracer items for providers' competence. In Namibia and Rwanda, providers' competence subdomain score was based on 15 tracer items instead of 17 as data were not available for the two tracer items: 1) provider’s advice on early/immediate breastfeeding, and 2) provider asked if the child received any deworming medications. In

For peer review only - http://bmjopen.bmj.com/site/about/guidelines.xhtml

Uganda, provider competence summary score was based on 14 items only as three tracer items were missing: 1) provider's advice on early/immediate breastfeeding, 2) provider asked if the child received any deworming medications, and 3) the provider checked palms / conjunctiva / mouth for pallor. Meanwhile, all countries had 12 tracer items for patient experience except Namibia where clients' reports on the assessment of child eating habits by a provider were missing. Stata 17 and R were used to analyze data and generate figures.

Patient and public involvement

This study relied on secondary analysis of publicly available datasets. Therefore, patients and the public were not involved in the formulation of this research.

Table 1: List of patients' experience and provider competence tracer activities

Quality Subdomain	Thematic area	Tracer activities	SPA tool
Patients' experience	Maternal	1. Client knows at least one side effect of iron<br>2. Provider encouraged questions<br>3. Provider advised 6 months exclusive breastfeeding<br>4. Provider ever advised breastfeeding<br>5. Provider explained iron side effects in any ANC visit<br>6. Provider explained how to take iron/folate in any ANC visit<br>7. Clients reported being advised on nutrition during pregnancy	Client exit interview - Antenatal care
	Child	1. Provider asked about child's usual eating habits<br>2. Child was weighed at the facility (exit)<br>3. Provider counseled on appropriate fluid/breastfeeding<br>4. Provider counseled on appropriate solid food intake<br>5. Provider discussed child's weight and growth	Client exit interview-Curative childcare and growth monitoring services
Provider competence	Maternal	1. Provider advised exclusive breastfeeding<br>2. Provider advised early/immediate breastfeeding<br>3. Provider described how to take iron pills<br>4. Provider mentioned side effects of iron<br>5. Provider explained purpose of iron pills<br>6. Provider discussed nutrition during pregnancy<br>7. Provider checked pallor of conjunctiva/palms for pallor<br>8. Provider measured weight during ANC	Observation checklist- Antenatal care
	Child	1. Provider counselled on child weight / growth chart<br>2. Provider weighed child<br>3. Provider plotted weight on growth chart<br>4. Provider checked palms / conjunctiva / mouth for pallor<br>5. Provider asked / caretaker mentioned if child unable to drink or breastfeed<br>6. Provider asked about normal (breast)feeding pattern<br>7. Provider asked about (breast)feeding pattern during this illness<br>8. Provider asked if child received any deworming<br>9. Provider asked if child received vitamin A within 6 months	Observation checklist- Curative childcare and growth monitoring services

For peer review only - http://bmjopen.bmj.com/site/about/guidelines.xhtml

Results

The present study included information on the quality of nutrition services in 18644 ANC visits and 23262 sick child visits in 8458 facilities across 130 subnational areas in 11 LMICS. More than half of the analyzed facilities (59 %, n=4990) were public, 76 % (n =6248) were classified as primary/non-hospital, and 39% (n=3298) of the facilities with available data were located in rural areas (Table 2). For this study, analysis was limited to primary (non-hospital facilities). All the studied countries, except DRC, scored below 50 % in both patients' experience and provider competence (Figure 1). For example, based on the 12 tracer activities assessed for patient experience, 36 % of the interviewed clients (on average) reported being aware of the nutrition services received in PHC and confirmed provider effectiveness in delivering these services. Similarly, only about 31 % of the observed providers (on average) successfully and effectively delivered essential nutrition services in PHC based on the 17 tracer activities assessed for provider competence. Generally, patient experience summary scores were higher than provider competence scores in 7 out of the 11 studied countries. Exceptions were observed in Kenya, Namibia, Rwanda, and Uganda. However, in three out of the four countries (Namibia, Rwanda, and Uganda), provider competence score was calculated using fewer tracer items as indicated in the methods section. Furthermore, all four countries conducted the SPA survey prior to 2013 so the scores may not be comparable to recent surveys.

Table 2: Descriptive characteristics of the analyzed sample

	Primary* (%)	Public (%)	Rural (%)	Facilities (n)	Districts (n)	Regions (n)
Afghanistan	38	23	1	160	-	7
DRC	54	61	76	1,412	502	26
Haiti	87	34	62	1,033	34	10
Kenya	64	50	-	695	142	8
Malawi	89	48	68	1,060	28	3
Namibia	89	74	-	411	4	13
Nepal	73	78	-	992	75	5
Rwanda	92	57	-	538	-	5
Senegal	92	81	21	466	-	14
Tanzania	78	67	63	1,200	10	30
Uganda	76	71	-	491	-	9
**Total**	**76**	**59**	**39**	**8,458**	**1297**	**130**

*Primary level was defined as services provided in outpatient consultations in public and private non-hospital SPA facilities

Figure 1: Quality of nutrition services by subdomain score in each of the studied countries

Assessment of individual tracer items of patients' experience with maternal nutrition services revealed that more than half of clients were encouraged to ask nutrition questions during ANC visits in 9 out of the 11 studied countries. In addition, more than 70 % of clients in each of the studied countries reported being advised on taking iron supplements during pregnancy. However, less than one third (32 %) of patients reported being advised on iron side effects in each of the studies countries. In contrast, when asked in an exit interview, more than 45% of clients knew at least one side effect of iron in 7 out of the 11 countries assessed. In addition, less than 34 % of clients reported being advised on exclusive breastfeeding in each of the studied countries, and only in Malawi and Namibia, more than 50 % of clients were ever advised on breastfeeding during ANC (Table 3).

Patients' experience with child nutrition services was found to be significantly lagging compared to maternal services. In five out of the 11 countries analyzed, more than 69 % of clients reported weighing the child at the facility; however, in all the studied countries, less than 42% of clients reported that the child's weight and growth

For peer review only - http://bmjopen.bmj.com/site/about/guidelines.xhtml

were discussed. Moreover, in 8 out of the 10 countries with available data, less than 25 % of clients reported being asked about the child's eating habits. In addition, in the majority of studied countries (10 out of 11), at most, 40 % of clients were counseled on appropriate fluid intake or breastfeeding, and less than 40 % were counseled on appropriate solid food intake. The only exception was DRC, where almost all clients (99%) reported being advised on both appropriate fluid and appropriate solid food intake (Table 3).

Table 3: Tracer Items of patient experience with maternal and child nutrition services

Patients' experience tracer items		AF	DRC*	HT	KE*	MW	NM*	NP	RW*	SN	TZ	UG*
Maternal	Client knows at least one side effect of iron	70.1 (13.5)	48.8 (3.7)	74.8 (4.1)	45 (3.5)	73.6 (3.6)	25.4 (2.4)	18.7 (2.1)	7.8 (1.7)	80.1 (3.8)	59.5 (2.5)	21.4 (3.0)
	Provider explained iron side effects in any ANC visit	30.9 (9)	20.8 (1.6)	16.3 (1.7)	18.1 (2.8)	16.4 (1.4)	29.3 (2.6)	21.8 (2.2)	11.6 (2.4)	32.4 (2.9)	24.8 (1.4)	17.2 (3.1)
	Provider explained how to take iron/folate in any ANC visit	72.1 (8.4)	88 (1.3)	86 (1.5)	94.5 (1.7)	96.2 (0.6)	95 (1.2)	95.5 (1)	86.8 (2.9)	91.9 (1.5)	96.1 (0.6)	87.5 (0)
	Provider encouraged questions	23.9 (9.3)	53.5 (2)	59.7 (2.5)	67.5 (3.1)	79.3 (1.9)	64.4 (3.1)	35.7 (2.5)	56.7 (3.5)	69.9 (3.1)	77.6 (1.5)	59.9 (3.7)
	Provider advised 6 months exclusive breastfeeding	1.1 (1.5)	20.4 (1.4)	16 (1.5)	28.8 (2.6)	34 (1.7)	8.4 (1.5)	12.9 (1.7)	28.2 (2.7)	11.8 (1.8)	25.2 (1.4)	16.3 (2.7)
	Provider ever advised breastfeeding	3.6 (2.8)	28.1 (1.6)	27.6 (1.9)	33.1 (2.6)	52.7 (1.7)	61.1 (2.9)	21.2 (2.1)	32.3 (2.8)	14.5 (1.9)	34.6 (1.5)	28.7 (3.4)
	Provider advised on nutrition during pregnancy	63.3 (8.2)	37.9 (1.7)	45.9 (2.2)	51.8 (3)	63.3 (1.9)	60 (2.9)	77.3 (2.2)	37.8 (2.8)	52.7 (3.3)	43.4 (1.6)	50.1 (3.7)
Child	Provider discussed child's weight and growth	39.5 (6.2)	33 (1.8)	34.2 (1.6)	22.8 (1.9)	16.1 (1)	33.5 (1.9)	16.2 (1.4)	42.3 (2.1)	37.6 (2.8)	37.8 (1.3)	20 (2.2)
	Provider counseled on appropriate solid food intake	38.6 (5.8)	99.6 (0.2)	15.4 (1.2)	16.5 (1.7)	8.8 (0.7)	21.7 (1.6)	18.2 (1.5)	10.8 (1.1)	37.7 (2.8)	12.2 (0.8)	24.6 (2.4)
	Provider counseled on appropriate fluid/breastfeeding	38.9 (5.4)	99.7 (0.2)	25 (1.4)	19.7 (1.7)	12.4 (0.8)	25.6 (1.6)	26.6 (1.6)	12.3 (1.2)	30.9 (2.7)	19.1 (1)	40 (2.8)
	Provider asked about child's usual eating habits	32 (5.2)	22 (1.5)	25.3 (1.4)	24.8 (2)	11.8 (0.8)	-	12.5 (1.2)	9.9 (1.1)	37.9 (2.8)	22.0 (1.0)	18.2 (2.1)
	Child was weighed at the facility (exit)	49.5 (7.8)	75.9 (1.7)	86.9 (1.3)	44.7 (2.5)	29.2 (1.5)	88.4 (1.6)	54.0 (2.0)	69.4 (1.9)	94.6 (1.3)	31 (1.3)	46.5 (3.0)

Note: -No data is available, Standard errors are in parentheses. AF: Afghanistan 2018, DRC: democratic republic of Congo 2018, HT: Haiti 2017, KE: Kenya 2010, MW: Malawi 2013, NM0: Namibia 2009, NP: Nepal 2015, RW: Rwanda 2007, SN: Senegal 2017, TZ: Tanzania 2015, UG: Uganda 2007. *Countries with older SPA surveys (prior to 2013 so data may not be comparable. Analysis is limited to primary(non-hospital) health facilities. Maternal experience was only assessed in facilities offering antenatal care services. Child experience was assessed in facilities offering services for under-five children.

Assessment of tracer activities for providers' competence in maternal nutrition services revealed that weight was measured during the ANC consultation for the majority (more than 70 %) of clients in 10 out of the 11 analyzed countries. The only exception was in Afghanistan, where only 26 % of observed providers measured the mother's weight. Signs of anemia were also checked by more than half of providers in 8 out of the 10 countries with

available data. However, in 7 out of the 11 countries analyzed, less than half of observed providers explained the purpose of iron or how to take iron pills. Furthermore, less than 15 % of providers mentioned the side effects of iron in all the studied countries. Counseling on breastfeeding was also one of the areas with significant gaps. Specifically, less than 20 % of observed providers advised on early/ immediate breastfeeding in all countries with available data (8 countries), and less than 35 % of observed providers advised exclusive breastfeeding in all the 11 studied countries (Table 4).

Table 4: Providers' competence in maternal and child nutrition services

Provider competence tracer items		AF	DRC*	HT	KE*	MW	NM*	NP	RW*	SN	TZ	UG*
Maternal	Provider checked pallor of conjunctiva/palms for pallor	28.4 (8.3)	56 (1.8)	51.8 (2.4)	79.9 (2.7)	78.1 (1.9)	58.9 (3.3)	36.8 (2.5)	78.5 (2.9)	88.1 (1.9)	54.5 (1.8)	-
	Provider described how to take iron pills	49.5 (9.7)	37.5 (1.8)	37 (2.3)	42.6 (3.3)	58.6 (2.3)	65.8 (2.8)	24.3 (2.1)	35.6 (3.3)	50.2 (3.1)	58.7 (1.7)	48.8 (3.7)
	Provider explained purpose of iron pills	47.8 (9.4)	29 (1.7)	30.2 (2.2)	39 (3.2)	57.4 (2.3)	53.5 (3.1)	21.6 (2.2)	31.6 (3.2)	61 (3.1)	59.7 (1.7)	31.4 (3.6)
	Provider mentioned side effects of iron	16.2 (8.4)	4.3 (0.7)	3.4 (0.8)	11.7 (2.2)	7.3 (1.1)	12.0 (2.0)	4.4 (1.0)	13.3 (2.3)	9.3 (1.7)	9.7 (1.0)	5.7 (1.9)
	Provider advised exclusive breastfeeding	0 (0)	10.8 (1.2)	5.3 (1.1)	23.4 (2.7)	5.5 (1.1)	34.4 (3.2)	1.5 (0.7)	20.5 (3)	4.2 (1.2)	11.6 (1.1)	15.9 (2.8)
	Provider advised early/immediate breastfeeding	1.8 (3.0)	7.2 (1.0)	2.2 (0.7)	18.1 (2.5)	3.2 (0.8)	-	2.7 (0.9)	-	5.2 (1.2)	7.0 (0.8)	-
	Provider discussed nutrition during pregnancy	58.4 (9)	28 (1.7)	50.2 (2.3)	45.3 (3.2)	35.3 (2.3)	46.3 (3.2)	55.9 (2.5)	40.3 (3.4)	54.6 (3.1)	31.4 (1.5)	32.6 (3.5)
	Provider measured weight during ANC	25.4 (8.9)	74.5 (1.8)	86.1 (1.8)	92.6 (1.8)	75.5 (2.2)	93.5 (1.8)	76.2 (2.3)	98.9 (0.6)	92.7 (1.3)	79.6 (1.5)	73.8 (3.9)
Child	Provider weighed child	47.5 (7.4)	72.7 (1.8)	83 (1.4)	42.2 (2.5)	17.6 (1.3)	88.1 (1.7)	45.5 (2.0)	63.4 (2.2)	94.3 (1.3)	17.1 (1.1)	45.3 (3.0)
	Provider plotted weight on growth chart	3.4 (1.7)	5.5 (0.9)	9.7 (1.1)	21.2 (1.8)	3.9 (0.6)	48.6 (2.4)	19.4 (1.6)	8.5 (1.1)	54.9 (3.2)	4.9 (0.6)	12.9 (2)
	Provider counselled on child weight / growth chart	13.8 (4.9)	9.6 (1.1)	8.4 (0.9)	28.2 (2)	10.9 (0.9)	30.2 (2)	4.9 (0.8)	16.4 (1.6)	11.4 (1.9)	24.7 (1.2)	8.8 (1.5)
	Provider asked if child received vitamin A within 6 months	4.3 (3.4)	7.2 (0.9)	2.9 (0.6)	21.9 (1.8)	2.2 (0.4)	30.7 (1.9)	0.8 (0.2)	16.7 (1.6)	32.3 (2.8)	2.7 (0.4)	9.3 (1.6)
	Provider asked if child received any deworming	0.3 (0.5)	11.7 (1.2)	2.9 (0.6)	20.2 (1.7)	2.4 (0.4)	-	3.5 (0.7)	-	28.3 (2.6)	4.7 (0.5)	-
	Provider checked palms / conjunctiva / mouth for pallor	32.3 (6.6)	72.3 (1.7)	48.1 (1.8)	57.1 (2.5)	46.9 (1.5)	39.1 (2.2)	14.5 (1.3)	35.6 (2)	18.1 (2.1)	39.6 (1.4)	72 (2.5)
	Provider asked / caretaker mentioned if child unable to drink or breastfeed	13.7 (5.2)	23.5 (1.5)	21.3 (1.4)	49.4 (2.3)	28.2 (1.3)	43.3 (2)	22.9 (1.6)	25.5 (1.5)	2.7 (0.8)	28.5 (1.2)	59.8 (2.7)

For peer review only - http://bmjopen.bmj.com/site/about/guidelines.xhtml

	Provider asked about normal (breast)feeding pattern	17.8 (5.7)	32.6 (1.8)	38.8 (1.7)	42.9 (2.3)	16.8 (1.1)	43.7 (2.1)	15.9 (1.4)	30.3 (1.8)	26.2 (2.4)	28.3 (1.2)	44.6 (2.8)
	Provider asked about (breast)feeding pattern during this illness	14.4 (5.6)	27.5 (1.7)	30.5 (1.6)	45.8 (2.2)	17.4 (1.1)	45.2 (2.1)	15.7 (1.3)	44.8 (2)	6.3 (1.2)	18.5 (1.0)	39.6 (2.8)

Note: -No data is available, standard errors are in parentheses. AF: Afghanistan 2018, DRC: democratic republic of Congo 2018, HT: Haiti 2017, KE: Kenya 2010, MW: Malawi 2013, NM0: Namibia 2009, NP: Nepal 2015, RW: Rwanda 2007, SN: Senegal 2017, TZ: Tanzania 2015, UG: Uganda 2007. *Countries with older SPA surveys (prior to 2013 so data may not be comparable. Analysis is limited to primary(non-hospital) health facilities. Competence of providers in Maternal services was only assessed in facilities offering antenatal care services. Competence of providers Child services was assessed in facilities offering services for under-five children.

Similarly, measuring weight or checking for signs of anemia during sick child consultations were among the highest-scored tracer activities of provider competence. However, less than one-third (30%) of observed providers counseled clients on child growth in each of the studied countries and less than one quarter (21%) plotted the child weight on a growth chart in 9 out of the 11 countries studied. In addition, less than 45% of observed providers in each of the analyzed countries asked about the child's normal feeding pattern or feeding pattern during illness. Furthermore, in 8 out of the 11 countries analyzed, less than 30 % of observed providers asked whether the child was unable to feed or drink during sick child consultations. Also, whenever data was available, 32 % of providers, at most, asked if the child received vitamin A or a deworming medication in each of the studied countries (Table 4).

Discussion

Overall, the descriptive analysis of patients' experience and provider competence in the present study showed the delivery of low-quality maternal and child nutrition services at the PHC level across 11 LMICs, where only one country (DRC) reported a provider competence score above 50%. These findings highlight the urgent need to integrate quality essential and preventive nutrition services into routine PHC in LMICs.

We also found compelling evidence indicating the lack of depth in providers' assessment of the nutritional status of expectant mothers and children. Across all assessed countries, providers commonly took anthropometric measurements of expectant mothers and children; however, such assessments were not always followed up with advice or counseling about growth patterns, developmental milestones, or dietary advice which is critically important for mitigating both undernutrition and overweight. The WHO child growth standard also recommends that children's weight and height are plotted on the standard growth chart following a physical examination, as they can be good predictors of dietary status and future disease risk when followed over time (16).

Explaining side effects of iron by providers was also one of the most sub-optimally performed activities from both the patient and the provider perspectives. Iron supplements are required to be taken daily, and empirical research shows that taking iron supplements is often accompanied by side effects such as constipation, cramps, etc., making treatment adherence difficult(17). It is important that providers are trained to forewarn anemic pregnant women of both the benefits and side-effects to attain treatment compliance and better health outcomes(17,18). Failure to counsel women on the proximal and long-lasting maternal, neonatal, and child health benefits of iron supplementation and breastfeeding during antenatal care represents a missed opportunity for improving maternal and child health(19). It is also important to consider that from a measurement perspective, many nutrition services and campaigns can be based in the community, which might explain the notably better performance in the patients' knowledge and awareness of Iron side effects, incongruent to the levels of provider competence in this analysis. Since the SPA data utilized in the present analysis only focuses on facility-based nutrition services, future research and tools should consider capturing the provision of community-based nutrition services.

For peer review only

This analysis also suggests that patient health literacy and confidence may be a determinant of receiving high-quality nutrition services. Positive patient perception of patient-provider interactions and quality of care is associated with better health experiences (20). Over 70 percent of women in two out of 11 countries reviewed showed that there was a lack of providers who encouraged women to ask questions during ANC. Positive provider interaction with mothers in the healthcare environment has been shown to be psychodynamic and therapeutic, thereby leading to greater patient satisfaction with the quality of care (21).To encourage question-asking behavior, it is important that health providers use simple and empathic language when dealing with expectant mothers and allow them to express themselves without fear (22).

Our study should be interpreted considering its limitations. First, we included a comprehensive sample of the latest available SPA surveys at the time of the analysis. Included data came from different geographic contexts and time periods, which might have introduced potential bias in the results. Specifically, countries with more recent SPA surveys could have benefited from updated knowledge and the recent advancements in nutrition services compared to countries with older SPA surveys. Our study is also descriptive in nature; primarily aiming at highlighting the quality of nutrition services at the PHC level in each of the studied countries rather than cross-country comparison. Therefore, we did not utilize inferential statistics to statistically test or account for the social and/or temporal variations across countries. In addition, we defined PHC as services provided in outpatient consultations in public and private non-hospital SPA facilities, while countries may vary in their definition of what constitute a PHC system. Meanwhile, data was not available for few tracer items in Namibia, Rwanda, and Uganda. However, missing data was less than 20 % of the total number of tracer items so these three countries were not dropped from the analysis. To minimize potential bias in the present findings, we reported the results for each individual tracer item per country whenever data was available. Future studies may utilize mixed-effect models that incorporate sociodemographic and temporal factors to determine whether the observed differences between countries were statistically significant.

Comprehensive SPA surveys continue to be essential tools in the assessment of health service delivery in LMICs. However, the surveys lack an array of vital evidence-based nutrition interventions such as calcium, balanced energy, and protein, multiple micronutrient supplementation in pregnancy. Up to our knowledge, there is no validation studies that document the validity and the reliability of the SPA tools/instruments in assessing the quality-of-service delivery of nutrition services at PHC level. Further, facility surveys generally lack specificity in topics key to nutrition assessment and training, especially in non-communicable disease services

Notably, our findings give rise to additional questions in understanding and framing the drivers influencing the provision of quality nutrition services in PHC. While the availability of nutrition services through investments in system inputs (drugs, diagnostics, equipment, workforce, and information systems) is a cornerstone for quality of care, service delivery processes such as having competent providers and clients who are engaged in their care is key to achieving the effective coverage for nutrition services (23,24). Future research should also consider the use of available service delivery indicators to understand the relationship between the quality of nutrition service delivery and nutrition-related health outcomes in LMICS.

Conclusion

Although provider competence and patients 'experience across the 11 assessed countries demonstrated the basic delivery of nutrition services, the apparent deficiency in the extent and depth of questions asked for the majority of assessed tracer activities reveal significant opportunities for improving the quality of nutrition service delivery process at the PHC level. Measuring the quality of nutrition services in PHC also remains a challenge due to the limited nature of some of the existing indicators; however, multiple opportunities exist for improving and utilizing the current tools.

Ethics statements

For peer review only - http://bmjopen.bmj.com/site/about/guidelines.xhtml

Data included in the present analysis were extracted from anonymized publicly available datasets and no patient consent was needed

Data availability statement The original datasets analyzed during the current study are available from the DHS program website, https://dhsprogram.com/data/available-datasets.cfm. The analytical dataset generated during the current study is available from the corresponding author on reasonable request

Contributors : MR,TBM and MVU were responsible for the conception of this study and for methods development. MR and TBM analysed data. MR, TBM, LO and CF prepared the first draft. MR, LO, CF, MVU all contributed to subsequent iterations of the draft. All authors reviewed results or reviewed and contributed to the final manuscript.

Funding: This research was supported, in whole or in part, by the Bill and Melinda Gates Foundation (Grant number INV000932), and it had no role in study design, data collection, data analysis, data interpretation or writing of the manuscript.

Competing interests: None declared.

For peer review only

For peer review only - http://bmjopen.bmj.com/site/about/guidelines.xhtml

References

1. UNICEF. 2020 Global Nutrition Report - Global Nutrition Report [Internet]. 2020 [cited 2020 Nov 2]. Available from: https://globalnutritionreport.org/reports/2020-global-nutrition-report/

2. Murray CJL, Aravkin AY, Zheng P, Abbafati C, Abbas KM, Abbasi-Kangevari M, et al. Global burden of 87 risk factors in 204 countries and territories, 1990–2019: a systematic analysis for the Global Burden of Disease Study 2019. The Lancet. 2020 Oct 17;396(10258):1223–49.

3. United Nations. Policy Brief: The Impact of COVID-19 on Food Security and Nutrition |Policy Support and Governance| Food and Agriculture Organization of the United Nations [Internet]. Un. 2020 [cited 2021 Apr 17]. Available from: http://www.fao.org/policy-support/tools-and-publications/resources-details/en/c/1287907/

4. World Bank. Food Security and COVID-19 [Internet]. Brief. 2021 [cited 2021 Apr 17]. Available from: https://www.worldbank.org/en/topic/agriculture/brief/food-security-and-covid-19

5. Kraef C, Wood B, Von Philipsborn P, Singh S, Peterson SS. Primary health care and nutrition. 2020.

6. America I of MC on Q of HC in. Crossing the Quality Chasm: A New Health System for the 21st Century. J Healthc Qual. 2002;24(5):52.

7. Primary Health Care Performance Initiative. The PHCPI Conceptual Framework [Internet]. 2018 [cited 2020 Nov 28]. Available from: https://improvingphc.org/phcpi-conceptual-framework

8. Nutrition [Internet]. [cited 2022 Mar 26]. Available from: https://www.who.int/news-room/facts-in-pictures/detail/nutrition

9. Amouzou A, Leslie HH, Ram M, Fox M, Jiwani SS, Requejo J, et al. Advances in the measurement of coverage for RMNCH and nutrition: from contact to effective coverage. BMJ Glob Health. 2019 May 1;4(Suppl 4):e001297.

10. Gillespie S, Menon P, Heidkamp R, Piwoz E, Rawat R, Munos M, et al. Measuring the coverage of nutrition interventions along the continuum of care: time to act at scale. BMJ Glob Health. 2019;4(Suppl 4):e001290.

11. King SE, Sheffel A, Heidkamp R, Xu YY, Walton S, Munos MK. Advancing nutrition measurement: Developing quantitative measures of nutrition service quality for pregnant women and children in low- and middle-income country health systems. Matern Child Nutr. 2022 Jan;18(1):e13279.

12. Mallick L, Temsah G, Benedict RK. Facility-based nutrition readiness and delivery of maternal and child nutrition services using service provision assessment surveys. 2018 Sep 1 [cited 2022 Mar 26]; Available from: https://dhsprogram.com/publications/publication-cr49-comparative-reports.cfm

13. Marsh AD, Muzigaba M, Diaz T, Requejo J, Jackson D, Chou D, et al. Effective coverage measurement in maternal, newborn, child, and adolescent health and nutrition: progress, future prospects, and implications for quality health systems. Lancet Glob Health. 2020 May;8(5):e730–6.

14. USAID. The DHS Program - Service Provision Assessments (SPA) [Internet]. Demographic Health Surveys. 2019 [cited 2020 Nov 28]. p. 1–2. Available from: https://dhsprogram.com/What-We-Do/Survey-Types/SPA.cfm

For peer review only

For peer review only - http://bmjopen.bmj.com/site/about/guidelines.xhtml

15. SPA Questionnaires 2022 (English) [Internet]. [cited 2022 Nov 27]. Available from: https://dhsprogram.com/publications/publication-SPAQ8-SPA-Questionnaires-and-Manuals.cfm

16. Child growth standards [Internet]. [cited 2022 Mar 26]. Available from: https://www.who.int/tools/child-growth-standards

17. Christian P, Mullany LC, Hurley KM, Katz J, Black RE. Nutrition and maternal, neonatal, and child health. Semin Perinatol. 2015 Aug;39(5):361–72.

18. Smith ER, Shankar AH, Wu LSF, Aboud S, Adu-Afarwuah S, Ali H, et al. Modifiers of the effect of maternal multiple micronutrient supplementation on stillbirth, birth outcomes, and infant mortality: a meta-analysis of individual patient data from 17 randomised trials in low-income and middle-income countries. Lancet Glob Health. 2017 Nov;5(11):e1090–100.

19. Joseph NT, Piwoz E, Lee D, Malata A, Leslie HH. Examining coverage, content, and impact of maternal nutrition interventions: the case for quality-adjusted coverage measurement. J Glob Health. 10(1):010501.

20. Ardey R, Ardey R. Patient Perceptions and Expectations From Primary Health-care Providers in India. J Fam Med Prim Care. 2015 Mar;4(1):53–63.

21. Perla L. Patient compliance and satisfaction with nursing care during delivery and recovery. J Nurs Care Qual. 2002 Jan;16(2):60–6.

22. Redshaw M. Women as consumers of maternity care: measuring “satisfaction” or “dissatisfaction”? Birth Berkeley Calif. 2008 Mar;35(1):73–6.

23. Amouzou A, Leslie HH, Ram M, Fox M, Jiwani SS, Requejo J, et al. Advances in the measurement of coverage for RMNCH and nutrition: from contact to effective coverage. BMJ Glob Health. 2019;4:114–24.

24. Nguyen PH, Khuong LQ, Pramanik P, Billah SM, Menon P, Piwoz E, et al. Effective coverage of nutrition interventions across the continuum of care in Bangladesh: Insights from nationwide cross-sectional household and health facility surveys. BMJ Open. 2021 Jan 20;11(1):40109.

For peer review only

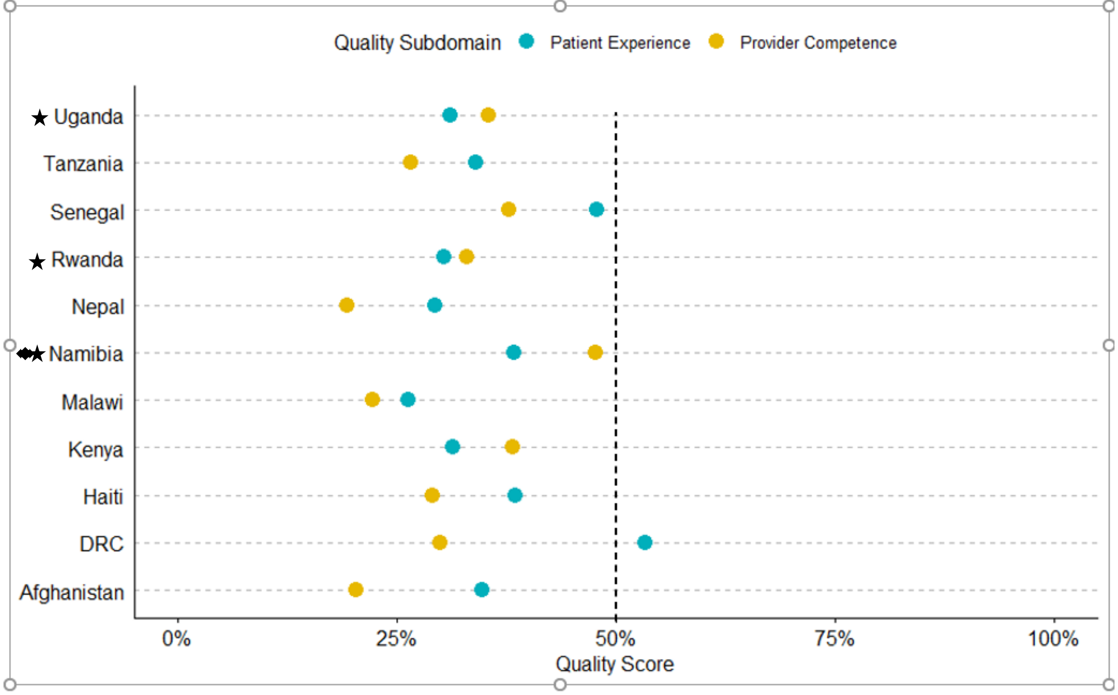

★Provider competence summary score is calculated as the average of 17 tracer items extracted from the Service Provision Assessment (SPA) survey in all 11 countries except Namibia, Rwanda, and Uganda. In Namibia and Rwanda, the provider competence subdomain was missing 2 tracer items (the provider advised early/immediate breastfeeding , the provider asked if the child received a deworming medication) and summary score was calculated as the average of the 15 remaining tracer items, and in Uganda, provider competence subdomain was missing 3 tracer items (the provider advised early/immediate breastfeeding , the provider asked if the child received a deworming medication, the provider checked for pallor for conjunctiva and/or pallor for palms)and the summary score was calculated as the average of the remaining 14 tracer items. Patient experience was calculated as the average of 12 tracer items extracted from the Service Provision Assessment (SPA) survey in all 11 countries except Namibia where clients' reports on the assessment of child eating habits by a provider were missing

Figure 1: Quality of nutrition services by subdomain score in each of the studied countries

The RECORD statement – checklist of items, extended from the STROBE statement, that should be reported in observational studies using routinely collected health data.

	Item No.	STROBE items	Location in manuscript where items are reported	RECORD items	Location in manuscript where items are reported
**Title and abstract**					
	1	(a) Indicate the study's design with a commonly used term in the title or the abstract (b) Provide in the abstract an informative and balanced summary of what was done and what was found	(a) line 21, page 2<br><br>(b) lines 19-39, page 2	RECORD 1.1: The type of data used should be specified in the title or abstract. When possible, the name of the databases used should be included.<br><br>RECORD 1.2: If applicable, the geographic region and timeframe within which the study took place should be reported in the title or abstract.<br><br>RECORD 1.3: If linkage between databases was conducted for the study, this should be clearly stated in the title or abstract.	1.1: line 22, page 2<br><br>1.2: lines 22-25, page 2<br><br>1.3: NA
**Introduction**					
Background rationale	2	Explain the scientific background and rationale for the investigation being reported	Lines 53-83, page 3		
Objectives	3	State specific objectives, including any prespecified hypotheses	Lines 84-88, page 3		
**Methods**					
Study Design	4	Present key elements of study design early in the paper	Lines 92, page 4		
Setting	5	Describe the setting, locations, and relevant dates, including periods of recruitment, exposure, follow-up, and data collection	Lines 92-101, page 4		

Participants	6	*(a) Cohort study* - Give the eligibility criteria, and the sources and methods of selection of participants. Describe methods of follow-up<br>*Case-control study* - Give the eligibility criteria, and the sources and methods of case ascertainment and control selection. Give the rationale for the choice of cases and controls<br>*Cross-sectional study* - Give the eligibility criteria, and the sources and methods of selection of participants<br><br>*(b) Cohort study* - For matched studies, give matching criteria and number of exposed and unexposed<br>*Case-control study* - For matched studies, give matching criteria and the number of controls per case	NA	RECORD 6.1: The methods of study population selection (such as codes or algorithms used to identify subjects) should be listed in detail. If this is not possible, an explanation should be provided.<br><br>RECORD 6.2: Any validation studies of the codes or algorithms used to select the population should be referenced. If validation was conducted for this study and not published elsewhere, detailed methods and results should be provided.<br><br>RECORD 6.3: If the study involved linkage of databases, consider use of a flow diagram or other graphical display to demonstrate the data linkage process, including the number of individuals with linked data at each stage.	6.1: NA- secondary analysis of publicly available anonymized data was used<br>6.2: NA- secondary analysis of publicly available anonymized data was used<br><br>6.3: NA- no linkage was done
Variables	7	Clearly define all outcomes, exposures, predictors, potential confounders, and effect modifiers. Give diagnostic criteria, if applicable.	Lines 103-116, page 5<br>Table 1-Page 6	RECORD 7.1: A complete list of codes and algorithms used to classify exposures, outcomes, confounders, and effect modifiers should be provided. If these cannot be reported, an explanation should be provided.	Lines 103-116, page 5<br>Table 1-Page 6<br><br>Confounders and effect modifiers were not measures in descriptive analysis – no regression models were carried out t
Data sources/ measurement	8	For each variable of interest, give sources of data and details	Lines 92-101<br>Table 1 -page 6		

For peer review only

		of methods of assessment (measurement). Describe comparability of assessment methods if there is more than one group			
Bias	9	Describe any efforts to address potential sources of bias	NA		
Study size	10	Explain how the study size was arrived at	Lines 92-101, page 5		
Quantitative variables	11	Explain how quantitative variables were handled in the analyses. If applicable, describe which groupings were chosen, and why	Lines 102-129, page 5		
Statistical methods	12	(a) Describe all statistical methods, including those used to control for confounding<br>(b) Describe any methods used to examine subgroups and interactions<br>(c) Explain how missing data were addressed<br>(d) *Cohort study* - If applicable, explain how loss to follow-up was addressed<br>*Case-control study* - If applicable, explain how matching of cases and controls was addressed<br>*Cross-sectional study* - If applicable, describe analytical methods taking account of sampling strategy<br>(e) Describe any sensitivity analyses	a) Lines 118-129, page 5<br>b) NA<br>c)Lines 126-129, page 5<br>d)NA<br>e)NA		

For peer review only

Data access and cleaning methods		..		RECORD 12.1: Authors should describe the extent to which the investigators had access to the database population used to create the study population.<br><br>RECORD 12.2: Authors should provide information on the data cleaning methods used in the study.	12.1: Data is publicly available (line 131, page 5) so section 12.1 does not apply<br><br>12.2: NA as access to already cleaned data was provided through DHS website
Linkage		..		RECORD 12.3: State whether the study included person-level, institutional-level, or other data linkage across two or more databases. The methods of linkage and methods of linkage quality evaluation should be provided.	12.3: NA
**Results**					
Participants	13	(a) Report the numbers of individuals at each stage of the study (*e.g.*, numbers potentially eligible, examined for eligibility, confirmed eligible, included in the study, completing follow-up, and analysed)<br>(b) Give reasons for non-participation at each stage.<br>(c) Consider use of a flow diagram	(a), (b),(c) are not applicable as the study is based on secondary data analysis	RECORD 13.1: Describe in detail the selection of the persons included in the study (*i.e.,* study population selection) including filtering based on data quality, data availability and linkage. The selection of included persons can be described in the text and/or by means of the study flow diagram.	13.1: lines 135-136, page 6
Descriptive data	14	(a) Give characteristics of study participants (*e.g.*, demographic, clinical, social) and information on exposures and potential confounders	Demographic of patients is not applicable; characteristics of population are described in lines		

For peer review only - http://bmjopen.bmj.com/site/about/guidelines.xhtml

		(b) Indicate the number of participants with missing data for each variable of interest<br>(c) *Cohort study* - summarise follow-up time (*e.g.*, average and total amount)	135-139, page 6 and Table 2, page 7		
Outcome data	15	*Cohort study* - Report numbers of outcome events or summary measures over time<br>*Case-control study* - Report numbers in each exposure category, or summary measures of exposure<br>*Cross-sectional study* - Report numbers of outcome events or summary measures	Lines 151-155, page 6		
Main results	16	(a) Give unadjusted estimates and, if applicable, confounder-adjusted estimates and their precision (e.g., 95% confidence interval). Make clear which confounders were adjusted for and why they were included<br>(b) Report category boundaries when continuous variables were categorized<br>(c) If relevant, consider translating estimates of relative risk into absolute risk for a meaningful time period	(a) Tables3-page 7 & Table 4-page 8<br>(b) NA<br>(c) NA		
Other analyses	17	Report other analyses done—e.g., analyses of subgroups and interactions, and sensitivity analyses	NA		
**Discussion**					
Key results	18	Summarise key results with reference to study objectives	Line 190-193, page 9		

For peer review only

Limitations	19	Discuss limitations of the study, taking into account sources of potential bias or imprecision. Discuss both direction and magnitude of any potential bias	Line 208-212, page 9<br>Lines 220-227, page 10	RECORD 19.1: Discuss the implications of using data that were not created or collected to answer the specific research question(s). Include discussion of misclassification bias, unmeasured confounding, missing data, and changing eligibility over time, as they pertain to the study being reported.	Line 208-212, page 9<br>Lines 220-227, page 10
Interpretation	20	Give a cautious overall interpretation of results considering objectives, limitations, multiplicity of analyses, results from similar studies, and other relevant evidence	Line 194-220, page 9-10		
Generalisability	21	Discuss the generalisability (external validity) of the study results	NA as all countries conducted a SPA survey were included in the present study		
**Other Information**					
Funding	22	Give the source of funding and the role of the funders for the present study and, if applicable, for the original study on which the present article is based	Lines 253-255, page 11		
Accessibility of protocol, raw data, and programming code		..		RECORD 22.1: Authors should provide information on how to access any supplemental information such as the study protocol, raw data, or programming code.	Lines 245-248, page 10

*Reference: Benchimol EI, Smeeth L, Guttmann A, Harron K, Moher D, Petersen I, Sørensen HT, von Elm E, Langan SM, the RECORD Working Committee. The REporting of studies Conducted using Observational Routinely-collected health Data (RECORD) Statement. *PLoS Medicine* 2015; in press.

*Checklist is protected under Creative Commons Attribution (CC BY) license.

For peer review only - http://bmjopen.bmj.com/site/about/guidelines.xhtml
